# Supplementary material for: Inhibition of PIKfyve prevents myocardial apoptosis and hypertrophy through activation of SIRT3 in obese mice
Source: EMBO Mol Med. 2017 Apr 10;9(6):770–85. doi: 10.15252/emmm.201607096 (PMC5452048; doi:10.15252/emmm.201607096)
Supplement: Supplementary file 1 — Appendix [file EMMM-9-770-s001.pdf]

Inhibition of PIKfyve prevents myocardial apoptosis and hypertrophy through activation of SIRT3 in obese mice

Helene Tronchere, Mathieu Cinato, Andrei Timotin, Laurie Guitou, Camille Villedieu, Helene Thibault, Delphine Baetz, Bernard Payrastre, Philippe Valet, Angelo Parini, Oksana Kunduzova and Frederic Boal

Appendix

Appendix Table S1

Appendix Table S1

Figure	p values	n per group
**1A**	p(H)=0.0022<br>p(H+STA)=0.0238	n=5;5;4;5
**1B**	p(2DG)=0,000327851<br>p(2DG+STA)=0,000985021	n=3;3
**1C**	p(H)=1,73497E-10<br>p(H+STA)=1,64667E-07	n=279;239;246;279
**1D**	p(H)=0,001598122<br>p(H+STA)=0,001754578	n=6;6;3;5
**1E-MitoSOX**	p(H)=1,50572E-09<br>p(H+STA)=7,45996E-07	n=30;25;16;22
**1E-MitoPY1**	p(H)=4,17123E-12<br>p(H+STA)=3,0993E-31	n=26;24;65;81
**1F-MitoSOX**	p(2DG)=2,24605E-37<br>p(2DG+STA)=3,23126E-38	n=154;85;159;142
**1F-MitoPY1**	p(2DG)=5,66723E-08<br>p(2DG+STA)=3,66877E-19	n=15;27;62;40
**2A**	p(H)=0,0066<br>p(H+STA)=0,0075	n=3;3;4;4
**2B**	p(H)=0,037316659<br>p(H+STA)=0,030044802	n=4;3;3;3
**2C**	p(2DG)=0,0002<br>p(2DG+STA)=0,0079	n=3;3;4;3
**2D**	p(2DG)=0,0005<br>p(2DG+STA)=0,0070	n=4;4;4;4
**2E**	p(H)=6,64015E-06<br>p(H+STA)=0,000112656	n=10;16;15;13
**2F**	p(2DG)=1,89082E-12<br>p(2DG+STA)=4,95436E-06	n=46;40;51;32
**2G**	p(H)=1,96997E-05<br>p(H+STA)=0,0003<br>p(H+Mdivi-1)=2,2448E-05	n=86;33;100;88;43
**2H**	p(2DG)=4,39527E-21<br>p(2DG+STA)=6,5583E-05<br>p(2DG+Mdivi-1)=0,00113866	n=86;41;107;114;52
**3B**	p(N+STA)=1,78309E-07<br>p(H+STA)=3,64902E-12	n=69;73;91;75
**3D**	p(N+STA)=0,000417432<br>p(H+STA)=0,001611735	n=3;3;3;3
**4B**	p(siCont+2DG)=1,00999E-11<br>p(siSIRT3+2DG)=2,68762E-15<br>p(siSIRT3+2DG+STA)=8,49542E-17<br>p(siCont+2DG vs siCont+2DG+STA)=9,41507E-08<br>p(siCont+2DG+STA vs siSIRT3+2DG+STA)=3,80429E-11	n=116;101;157;79;105;133;75;134
**4D**	p(siCont+2DG)=5,4264E-06	n=6;6;5;7;6;6;6;4

	p(siSIRT3+2DG)=6,26395E-07<br>p(siSIRT3+2DG+STA)=1,24641E-05<br>p(siCont+2DG vs siCont+2DG+STA)=8,18192E-05<br>p(siCont+2DG+STA vs siSIRT3+2DG+STA)=0,0005	
**5B**	p(ND vs. Vehicle-HFD)=0,00115386<br>p(Vehicle-HFD vs. STA-HFD)=1,57681E-06	n=6;8;5
**5C**	p(ND vs. Vehicle-HFD)=0,00056281<br>p(Vehicle-HFD vs. STA-HFD)=1,3975E-06	n=6;8;5
**5D**	p(ND vs. Vehicle-HFD)=8,08843E-05<br>p(Vehicle-HFD vs. STA-HFD)=0,000475435	n=6;8;5
**5E**	p(ND vs. Vehicle-HFD)=2,79186E-06<br>p(Vehicle-HFD vs. STA-HFD)=0,000279659	n=6;8;5
**5F**	p(ND vs. Vehicle-HFD)=0,0232<br>p(Vehicle-HFD vs. STA-HFD)=0,0431	n=5;4;5
**5G**	p(ND vs. Vehicle-HFD)=8,70066E-12<br>p(Vehicle-HFD vs. STA-HFD)=1,45491E-14	n=33;57;32
**5H**	p(ND vs. Vehicle-HFD)=0,0001<br>p(Vehicle-HFD vs. STA-HFD)=0,0168<br>p(ND vs. STA-HFD)=0,0014	n=6;4;6
**5I**	p(ND vs. Vehicle-HFD)=0,0020<br>p(Vehicle-HFD vs. STA-HFD)=0,0473<br>p(ND vs. STA-HFD)=0,0345	n=6;4;6
**5J**	p(ND vs. Vehicle-HFD)=0,000510233<br>p(Vehicle-HFD vs. STA-HFD)=0,007018196	n=7;5;5
**6B**	p(ND vs. Vehicle-HFD)=4,42196E-06<br>p(Vehicle-HFD vs. STA-HFD)=5,37517E-06	n=4;4;3
**6D**	p(ND vs. Vehicle-HFD)=<br>0,000415662<br>p(Vehicle-HFD vs. STA-HFD)=2,9604E-05	n=4;4;3
**6E**	p(ND vs. Vehicle-HFD)=0,0068<br>p(Vehicle-HFD vs. STA-HFD)=0,0026<br>p(ND vs. STA-HFD)=0,0399	n=3;5;6
**7B**	p(ND vs. Vehicle-HFD)=3,11528E-25<br>p(Vehicle-HFD vs. STA-HFD)=6,80775E-10<br>p(ND vs. STA-HFD)=7,96583E-09	n=4;4;6
**7D**	p(I, ND vs. Vehicle-HFD)=0,0447<br>p(I, Vehicle-HFD vs. STA-HFD)=0,0026	n=3;5;4
	p(II, ND vs. Vehicle-HFD)=0,0444<br>p(II, Vehicle-HFD vs. STA-HFD)=0,0204	n= 3;6;3
	p(III, Vehicle-HFD vs. STA-HFD)=0,0002	n=3;6;4
	p(IV-I, Vehicle-HFD vs. STA-HFD)=0,0085	n=4;6;4
	p(V, ND vs. Vehicle-HFD)=0,0230<br>p(V, Vehicle-HFD vs. STA-HFD)=0,000148371	n=4;6;4
**8A**	p=0,0089	n=4;3
**8B**	p=0,0036	n=4;3

**8C**	p=0,0153	n=4;3
**9B**	p(WT, ISO vs. C)=1,95534E-05<br>p(WT, ISO+STA vs.ISO)=0,004815233<br>p(SIRT3.KO, ISO vs.C)=0,013635739	n=9;3;4;9;3;4
**9C**	p=0,0102	n=3;4;3;4
**9D**	p(Vehicle vs. ISO)=0,0156<br>p(ISO vs. ISO+STA)=0,0232	n=4;3;4
**EV1B**	p=0,0095	n=3;3
**EV1C**	p(N vs. H)=0,0005<br>p(H vs. H+STA)=2,33398E-05<br>p(H vs. H+siPIKfyve)=0,000145237	n=27;41;54;65;22;42;40;49
**EV1D**	p(N vs. H)=0,0024<br>p(H vs. H+siPIKfyve)=0,0005	n=13;18;23;11
**EV1E**	p(N vs. H)=5,48656E-15<br>p(H vs. H+siPIKfyve)=4,43348E-15	n=44;35;53;48
**EV2**	p=1,11323E-06	n=4;4
**EV3A**	p=0,0007	n=6;6
**EV3B**	p=0,0475	n=5;5
**EV3D**	p=0,0471	n=5;5
**EV3E**	p=0,0063	n=6;5
