# Supplementary material for: Inhibition of PIKfyve prevents myocardial apoptosis and hypertrophy through activation of SIRT3 in obese mice
Source: EMBO Mol Med. 2017 Apr 10;9(6):770–85. doi: 10.15252/emmm.201607096 (PMC5452048; doi:10.15252/emmm.201607096)
Supplement: Supplementary file 2 — Expanded View Figures PDF [file EMMM-9-770-s002.pdf]

Expanded View Figures

Figure EV1. Silencing of PIKfyve recapitulates STA protective effects on mitochondria.

A H9C2 cells were treated with DMSO, STA or H_2O_2 for the indicated time and cell viability was assessed by MTT staining. Results are from three independent experiments.

B H9C2 cells were transfected with a control siRNA (siControl) or a siRNA targeting PIKfyve (siPIKfyve). PIKfyve mRNA level was measured by qRT-PCR. Student's test, $^{**}P < 0.01$ ($n = 3$).

C H9C2 cells were transfected as in (B), submitted to hypoxia (H) or kept in normoxia (N) in the presence of STA or DMSO alone. Mitochondrial ROS production was followed using MitoSOX. Scale bar is 10 μm. Quantification is shown on the right. Bonferroni's *post hoc* test: $^{***}P < 0.001$ between indicated conditions. ns, non-significant ($n = 22–65$).

D Cells transfected with a PIKfyve-siRNA were live-stained with MitoTracker to assess mitochondrial structure in normoxic (N) or hypoxic (H) conditions (left panel). Scale bar is 10 μm. Right panel shows quantification of mitochondrial fragmentation. Bonferroni's *post hoc* test: $^{*}P < 0.05$ and $^{***}P < 0.001$ between indicated conditions ($n = 11–23$).

E Cells were treated as in (D), fixed and stained for endogenous Drp1 to follow its oligomerization. Scale bar is 10 μm. Quantification of Drp1 particles per cell is shown on the right. Bonferroni's *post hoc* test: $^{***}P < 0.001$ between indicated conditions ($n = 35–53$).

Data information: Data are presented as mean ± SEM.

© 2017 The Authors

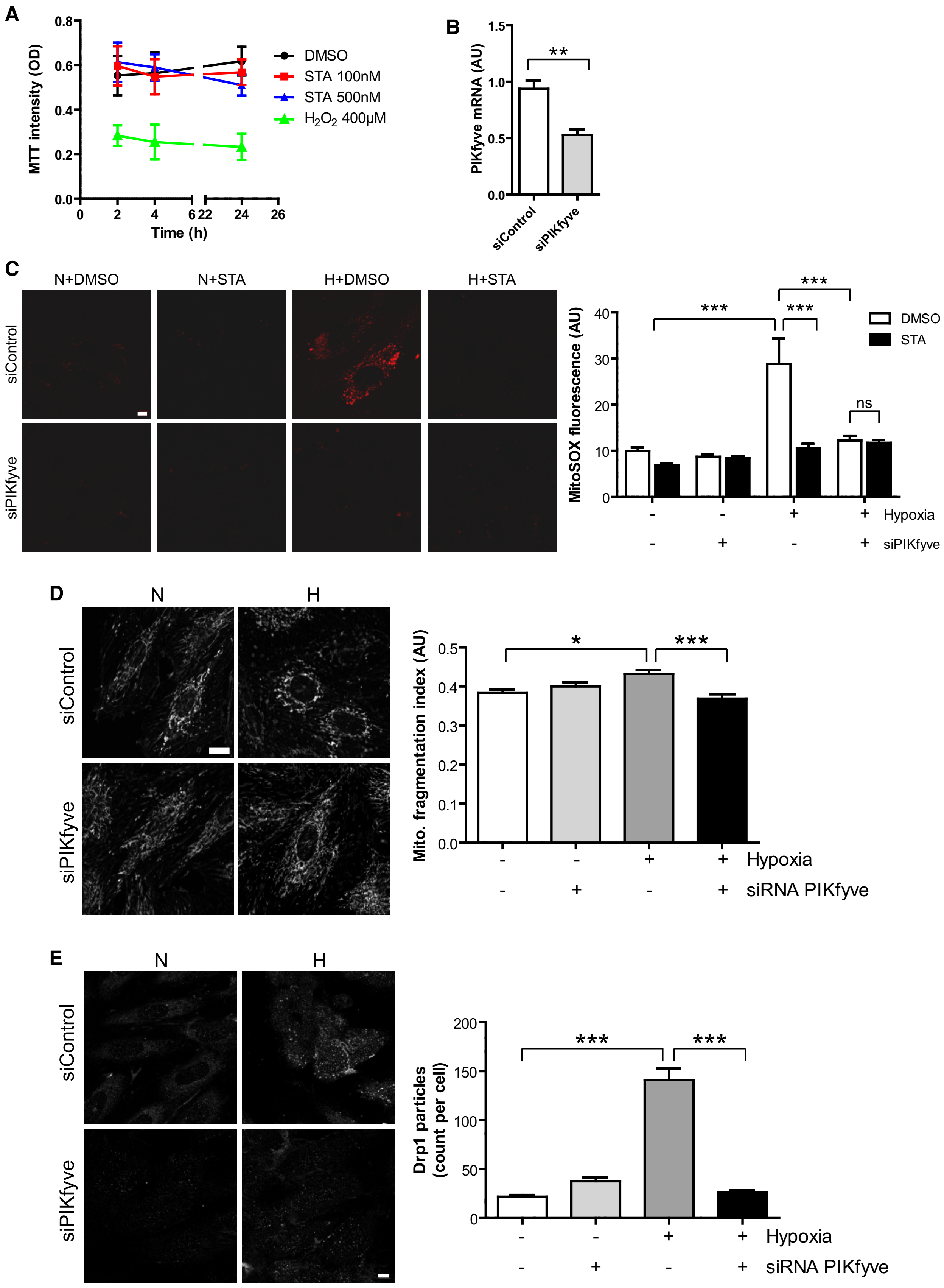

Figure EV1.

 © 2017 The Authors

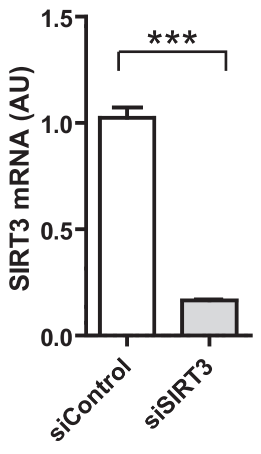

Figure EV2. Efficiency of SIRT3 siRNA.
HC92 cells were transfected with a control siRNA (siControl) or with a siRNA against SIRT3 (siSIRT3) and SIRT3 expression level was measured by qRT-PCR. Data are presented as mean ± SEM. Student's *t*-test, ***$P < 0.001$ ($n = 4$).

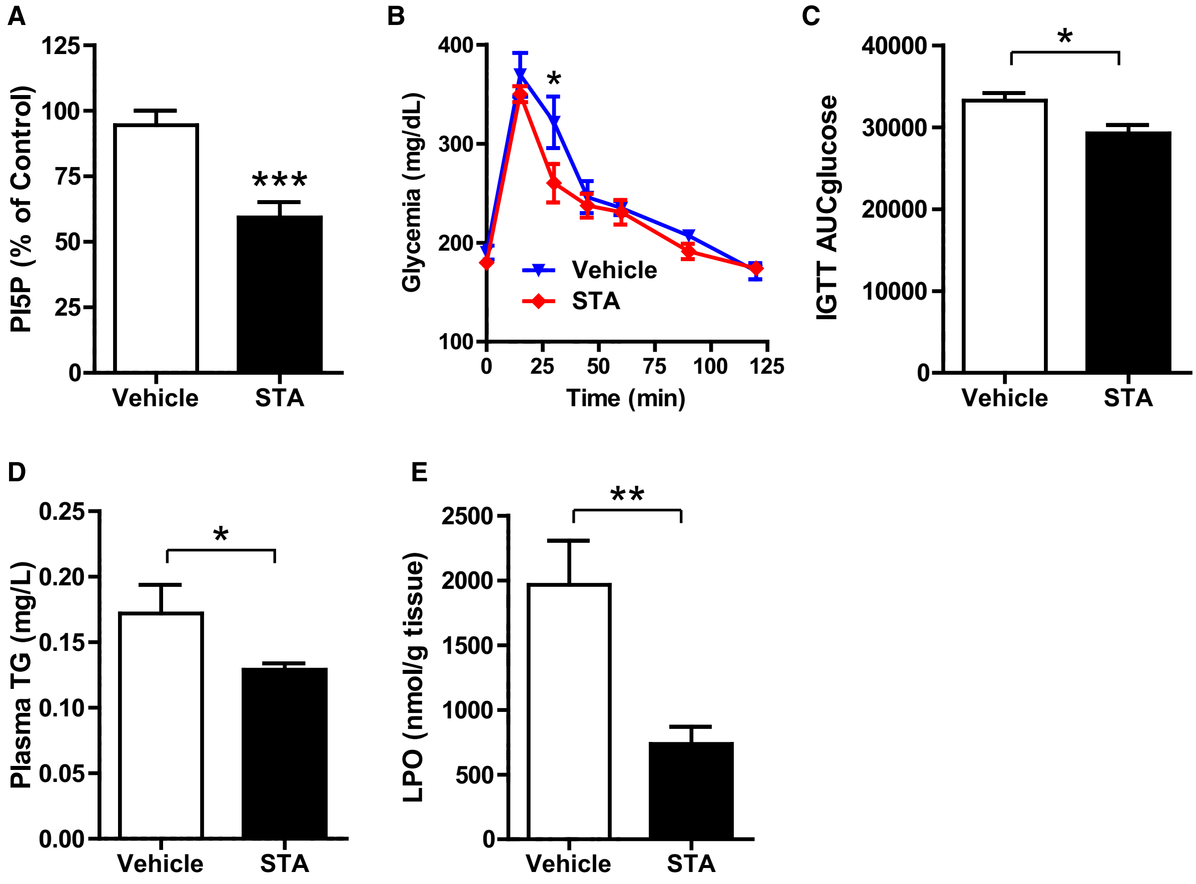

Figure EV3. Chronic STA treatment improves glucose tolerance and reduces plasma TG levels and cardiac LPO.

A PI5P levels were quantified in cardiac tissue by mass assay to monitor the efficacy of STA treatment in obese mice.
B Glucose tolerance test was performed on 6-h-fasted obese vehicle- or STA-treated mice.
C Area under the curve of glucose tolerance test from (B).
D Plasma triglycerides (TG) levels were quantified from vehicle- or STA-treated obese mice.
E Lipid peroxide (LPO) was quantified in cardiac tissue.

Data information: Data are presented as mean ± SEM. Student's *t*-test, ***$P < 0.001$, **$P < 0.01$, *$P < 0.05$. $n = 5$–6 mice per group.

© 2017 The Authors

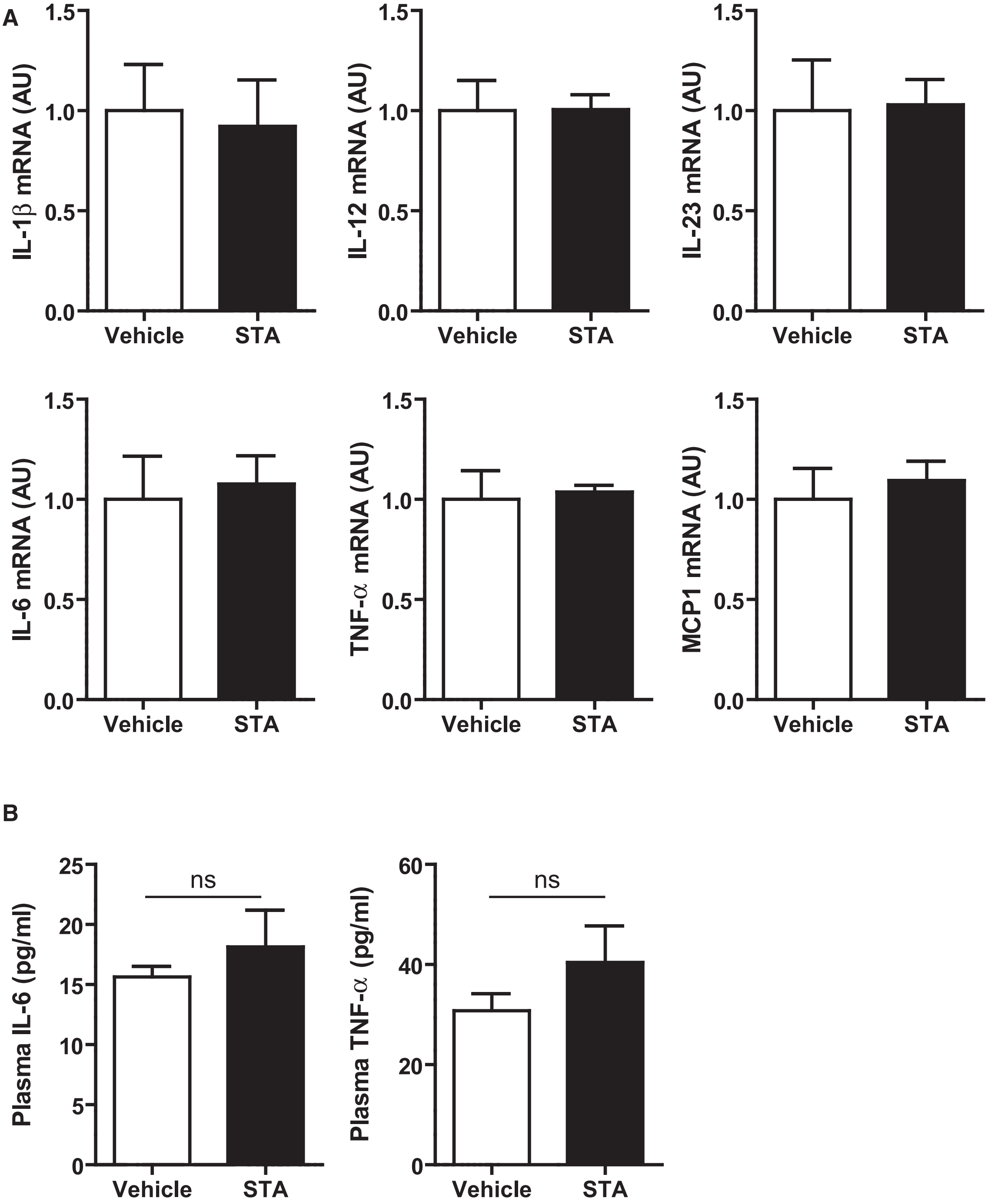

Figure EV4. Chronic STA treatment does not modify the expression and secretion of several inflammatory cytokines.

A Expression level of several inflammatory cytokines was quantified by qRT-PCR in cardiac tissue.

B Plasma IL-6 and TNF-α are not modified upon STA treatment in obese mice.

Data information: Data are presented as mean ± SEM. Student's *t*-test. ns, non-significant. $n = 5–6$ mice per group.

© 2017 The Authors
