# Supplementary material for: Inhibition of PIKfyve prevents myocardial apoptosis and hypertrophy through activation of SIRT3 in obese mice
Source: EMBO Mol Med. 2017 Apr 10;9(6):770–85. doi: 10.15252/emmm.201607096 (PMC5452048; doi:10.15252/emmm.201607096)
Supplement: Supplementary file 3 — Table EV1 [file EMMM-9-770-s003.docx]

**Table EV1. qRT-PCR primers used in this study**

| Target gene | Sense | Antisense |
| --- | --- | --- |
| PIKfyve | ATGGCCACAGATGACAAGAGTTCC | CAGACTGTGTTCTTGAAGGG |
| Mouse GAPDH | CTTTGTCAAGCTCATTTCCTGG | TCTTGCTCAGTGTCCTTGC |
| Rat GAPDH | TCCAGTATGACTCTACCCACG | CACGACATACTCAGCACCAG |
| Bax | CGGCGAATTGGAGATGAACT | GTCCACGTCAGCAATCATCCT |
| SIRT3 | ACAGCTACATGCACGGTCTG | ACACAATGTCGGGTTTCACA |
| Rat β-MHC | ACAACCCCTACGATTATGCG | CGCCTGTCAGCTTGTAAATG |
| Mouse β-MHC | AGGTGGCTCCGAGAAAGGAA | TGAGCCTTGGATTCTCAAACGT |
| BNP | GCACAAGATAGACCGGATCG | CCCAGGCAGAGTCAGAAAC |
| IL-12 | AGGTCACACTGGACCAAAGG | TGGTTTGATGATGTCCCTGA |
| IL-23 | GACTCAGCCAACTCCTCCAG | GGCACTAAGGGCTCAGTCAG |
| IL-1β | TCGCTCAGGGTCACAAGAAA | CATCAGAGGCAAGGAGGAAAAC |
| IL-6 | GCCCACCAAGAACGATAGTCA | CAAGAAGGCAACTGGATGGAA |
| TNF-α | TGGGACAGTGACCTGGACTGT | TTCGGAAAGCCCATTTGAGT |
| MCP1 | GCAGTTAACGCCCCACTCA | CCAGCCTACTCATTGGGATCA |
