# Supplementary figures and images for: Inhibition of PIKfyve prevents myocardial apoptosis and hypertrophy through activation of SIRT3 in obese mice
Source: EMBO Mol Med. 2017 Apr 10;9(6):770–85. doi: 10.15252/emmm.201607096 (PMC5452048; doi:10.15252/emmm.201607096)

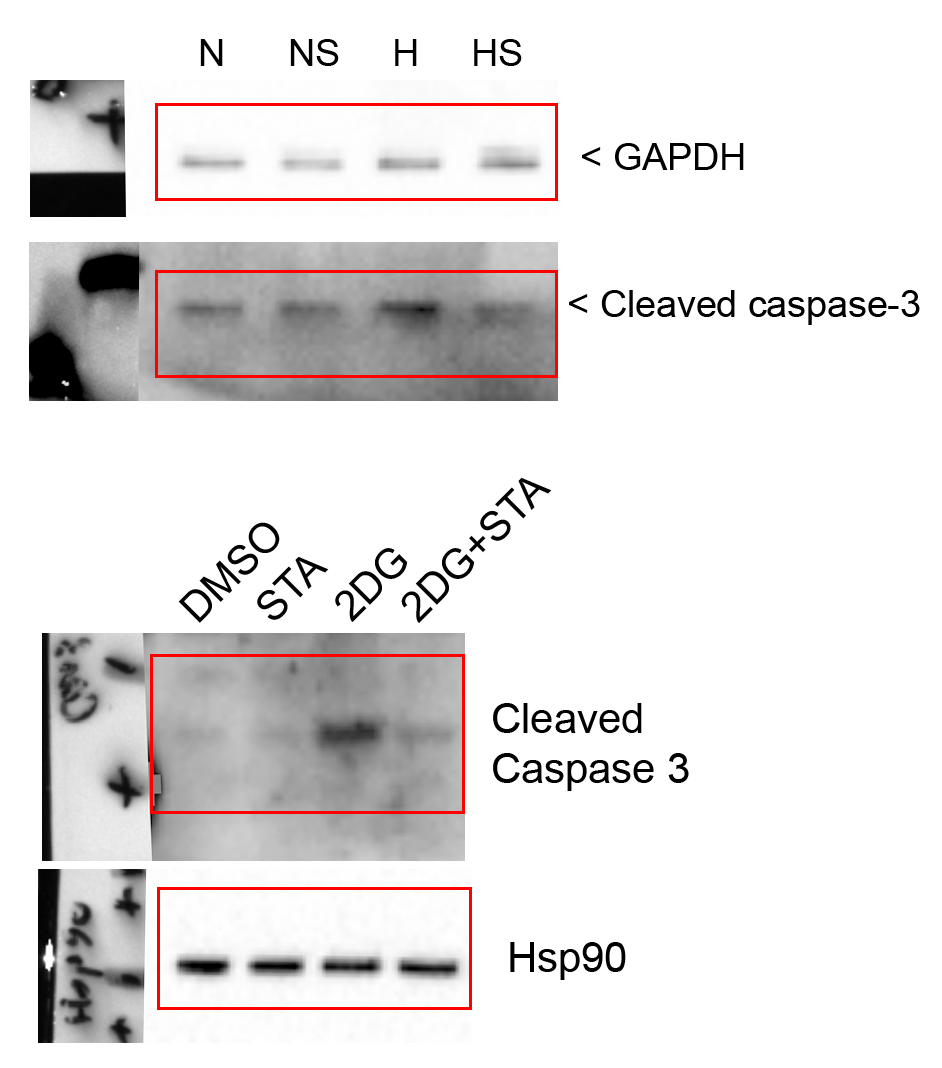

Supplement: Supplementary file 5 — Source Data for Figure 2 [file EMMM-9-770-s004.tif]

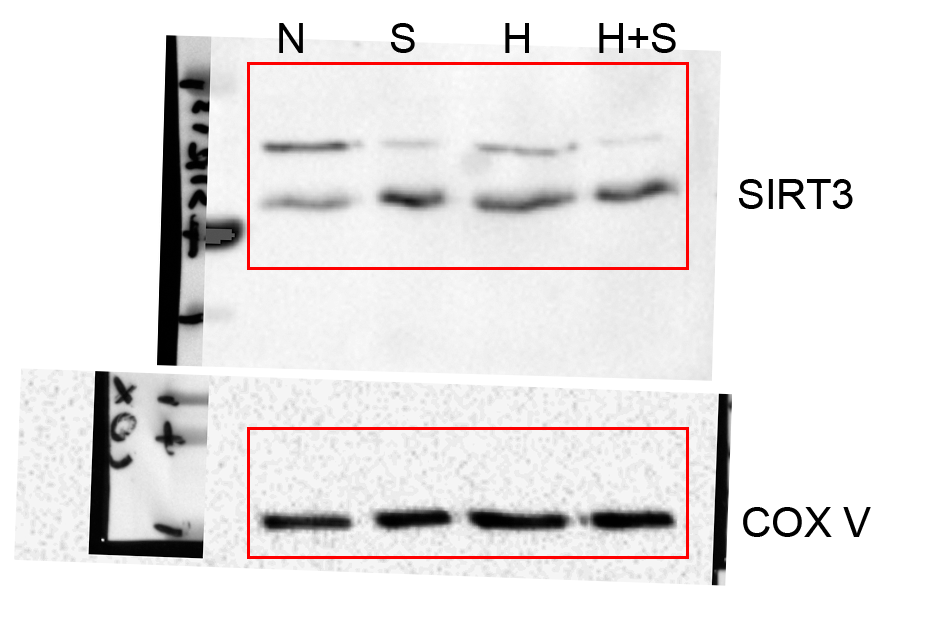

Supplement: Supplementary file 6 — Source Data for Figure 3 [file EMMM-9-770-s005.tif]

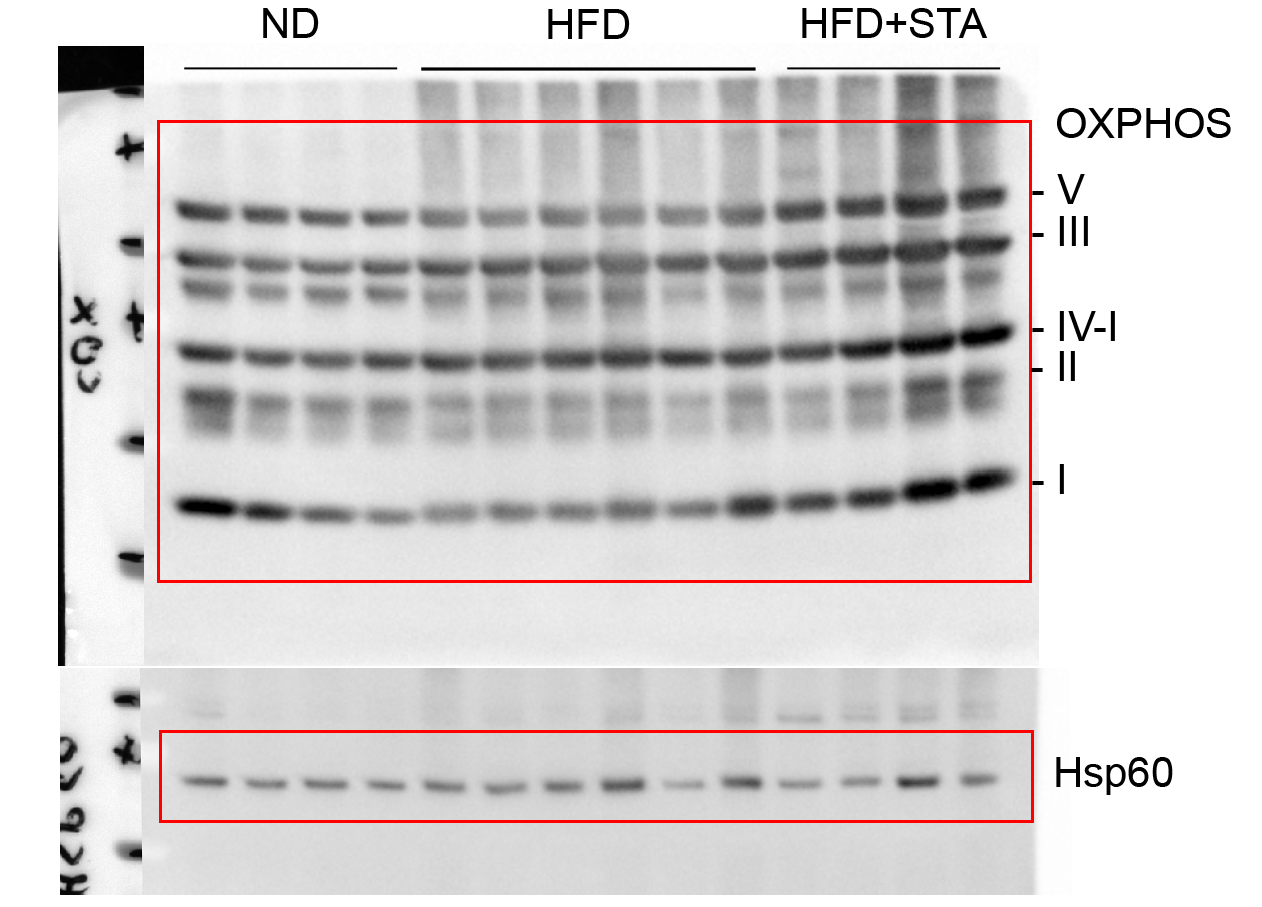

Supplement: Supplementary file 7 — Source Data for Figure 7 [file EMMM-9-770-s006.tif]

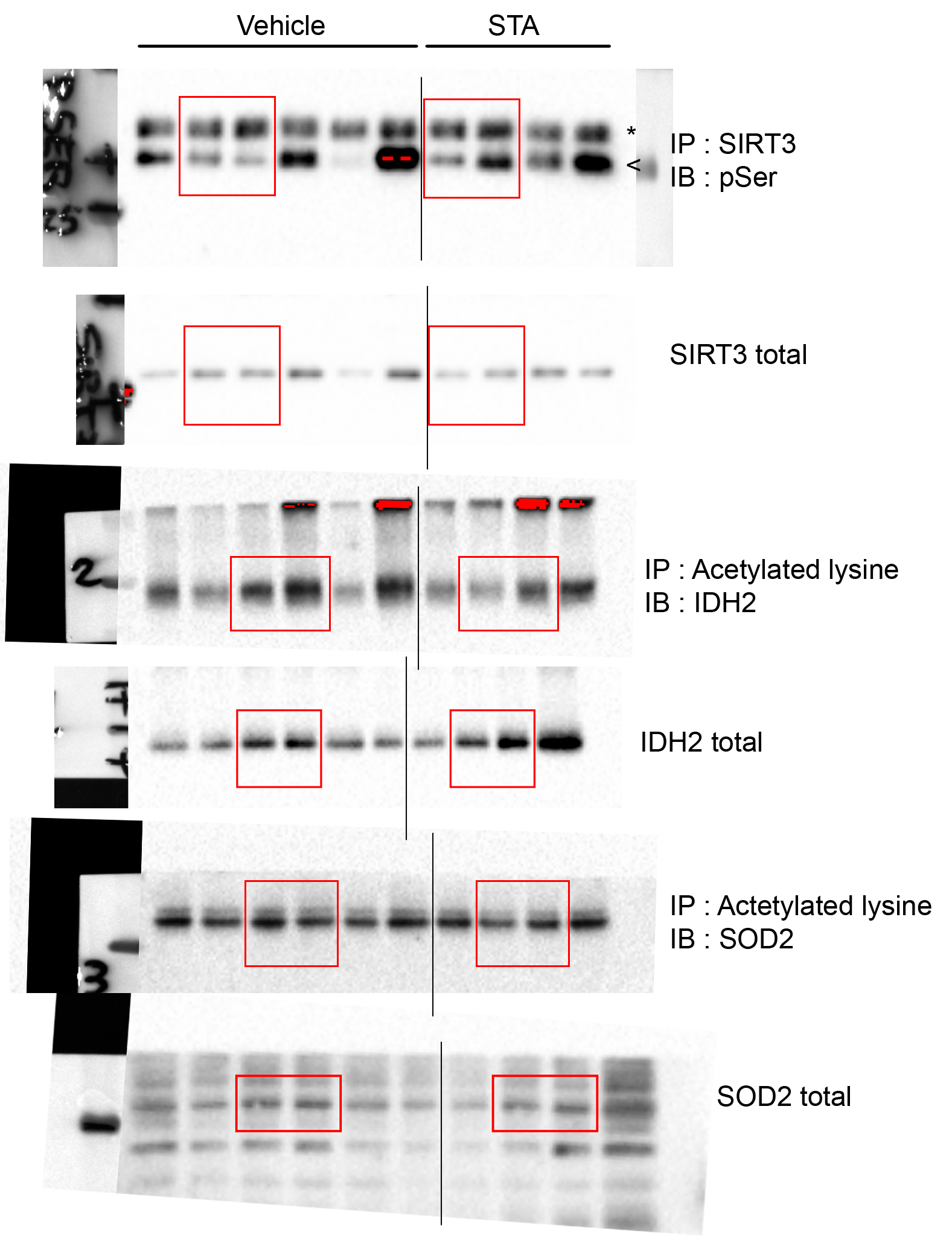

Supplement: Supplementary file 8 — Source Data for Figure 8 [file EMMM-9-770-s007.tif]

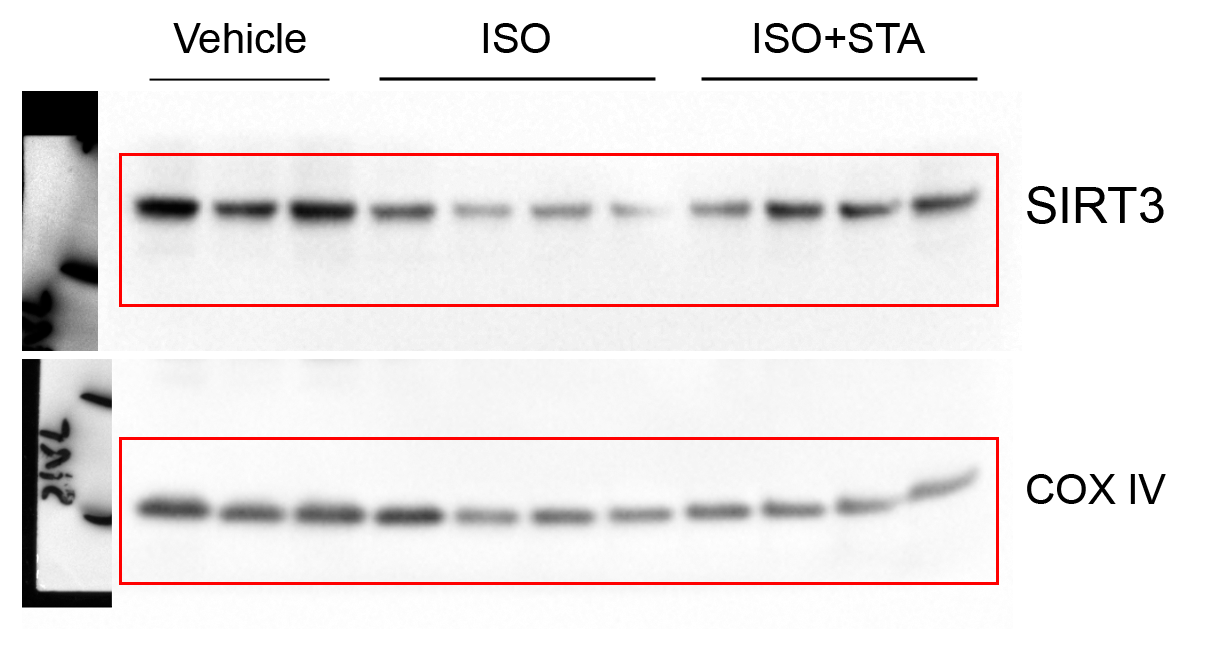

Supplement: Supplementary file 9 — Source Data for Figure 9 [file EMMM-9-770-s008.tif]
